# A Review on Cement Asphalt Emulsion Mortar Composites, Structural Development, and Performances

**DOI:** 10.3390/ma14123422

**Published:** 2021-06-21

**Authors:** Hussaini Abdullahi Umar, Xiaohui Zeng, Xuli Lan, Huasheng Zhu, Yirui Li, Hong Zhao, Haichuan Liu

**Affiliations:** 1 School of Civil Engineering, Central South University, Changsha 410075, China; lanxuli2020@csu.edu.cn (X.L.); julia74@126.com (H.Z.); liyirui2016@my.swjtu.edu.cn (Y.L.); zhaohong165@163.com (H.Z.); 13086611297@163.com (H.L.); 2 Department of Civil Engineering, Ahmadu Bello University, Zaria 810107, Nigeria

**Keywords:** damping performance, compressive strength, asphalt to cement ratio (A/C), mixing method, demulsification of emulsified asphalt

## Abstract

The use of cement emulsified asphalt mortar (CA mortar) in the track structure of high-speed speed railways has been gaining considerations by many researchers due to its coupled merits of the strength of cement as well as the flexibility of asphalt material. The asphalt to cement ratio (A/C) and the compatibility among constituent materials are crucial to the properties of CA mortar. To improve the performance properties and application of CA mortar, it is imperative to have a broad understanding of the composition mechanisms and compatibility between constituent materials. This paper summarizes interesting research outcomes related to the composition and properties of CA mortar. The consumption of water by cement promotes the breakdown of emulsified asphalt, likewise, the adsorption of asphalt droplets on the surface of cement grains retards the hydration process of cement. An appropriate A/C is required for the cement hydration rate to match the speed of demulsification of asphalt emulsion. Depending on the type and properties for which the CA mortar is designed to possess, the A/C ranges from 0.2 to 0.6 for type 1 (CAM I), and 0.6 to 1.2 for type 2 (CAM II). This paper also discusses measures taken to improve performance properties, compatibility, the interaction between constituent materials of CA mortar, and the use of additives as a partial replacement of cement in CA mortar production. The current review also suggests areas of interest for future research studies. This paper is useful to those who aim to understand or study the composition mechanisms and performance properties of CA mortar.

## 1. Introduction

With the design of high-speed rail, there has been an increasing demand for a high-quality railway system that includes railway tracks, communication, and a signal system, etc. Non-ballasted slab track is one of the foremost vital innovations created for high-speed railways. It is adopted all over the world due to the advantages it offers to the system over the ordinary ballasted track that includes reduced structural height, fewer maintenance requirements, durability, high lateral track resistance, which gives room for future speed increments, and no churning up of the ballast [1,2,3,4,5]. In mainland China, the China Railway Track System (CRTS), categorized into CRTS I, CRTS II, and CRTS III, are the main types of ballastless track systems adopted in recent decades. CRTS I and II track systems possess an almost similar structural setup, which is composed of rails, fasteners, a prefabricated concrete slab, an in-situ concrete trackbed, and an intermediary cushion layer. In the construction of CRTS I and II, a technique of top–down and bottom–up is endorsed; this technique shortens the time taken for installation and also controls the level and alignment of the high-speed railway operation [1]. As a result of this technique, it has become necessary to provide a narrow gap between the track slab and trackbed, which is filled up with a grouting material to serve as a cushion layer after hardening. cement emulsified asphalt mortar is used as the cushion material in the construction of CRTS I and II.

**Figure 1:**
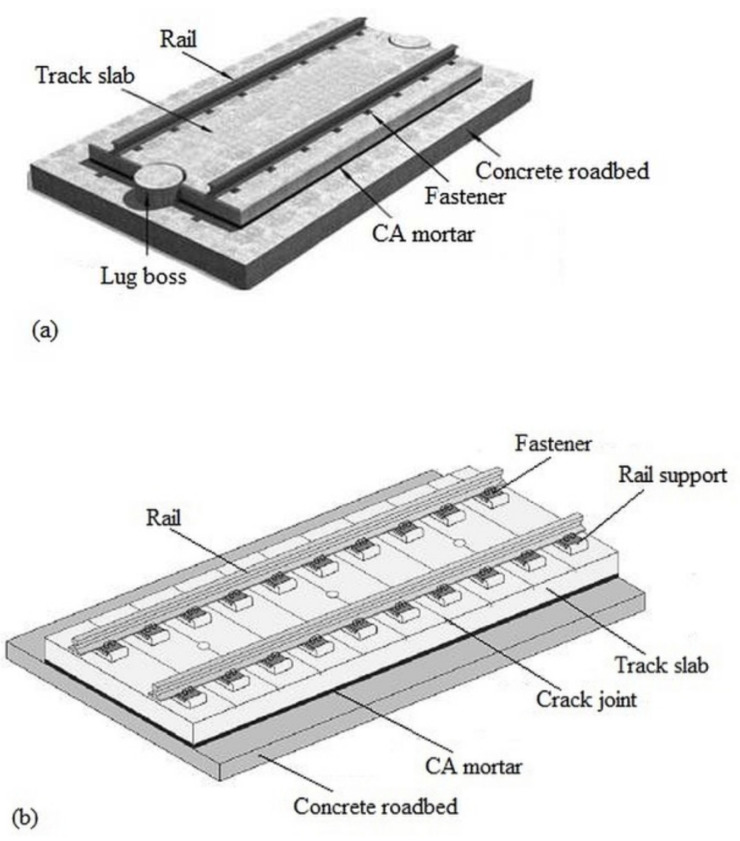
Structure of a slab ballastless track; (**a**) China Railway Track System (CRTS) I [7], (**b**) CRTS II [15]. CA: cement emulsified asphalt.

**Figure 2:**
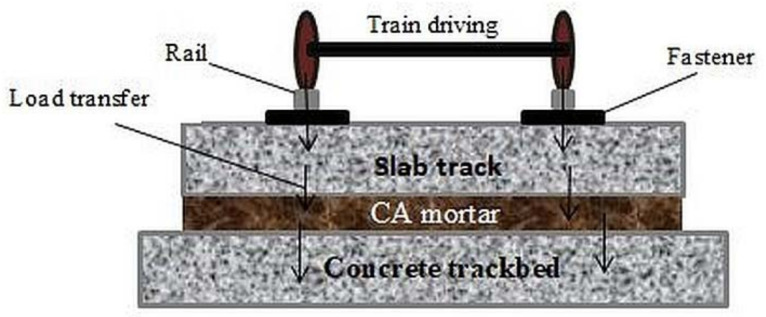
Schematic representation of layer arrangement and load transfer in the ballastless slab track system [12].

CA mortar (sometimes abbreviated to CAM) is one of the major construction materials for slab ballastless track in high-speed railways; it is an intermediate layer flung within the space between the track slab and the trackbed (as depicted in Figure 1) of CRTS I and CRTS II [3,6,7,8]. Cement and asphalt mortar is an organic–inorganic composite material primarily composed of asphalt emulsion, cement, sand, water, and other chemical admixtures [1,3,9]. This composite material possesses fascinating properties that are different from both concrete and asphalt material alone because it couples the strength of cement as well as the flexibility of asphalt material [10]. Being a structural member of the non-ballasted slab track, CA mortar offers tremendous advantages to the system which include providing support to the track and train, adjusting the track precision, facilitating load transfer (as illustrated in Figure 2), shock absorption, improving the damping ability of the track system and improving the riding comfort of high-speed rails [3,11,12]. CA mortar is produced by mixing its components using different mix proportions; its properties are mainly controlled by the proportion of asphalt to cement (A/C ratio), which is the ratio of the content of asphalt to the content of cement by mass or by volume; sometimes expressed as the ratio of asphalt emulsion to cement (AE/C). The addition of sand and a suitable amount of water ensures mixing stability and homogeneous distribution of CA mortar particles [13,14].

Two different types of CA mortar are used in the slab ballastless track system for high-speed rail, type I (CAM I), and type II (CAM II), which are used in the construction of CRTS I and CRTS II, respectively. CAM I is mainly characterized by having high asphalt content, less cement, and a higher A/C ratio ranging between 0.6 to 1.2; contrarily, CAM II has a high cement content with less asphalt content and an A/C of about 0.2 to 0.6 [1]. However, the properties and composition mechanisms of these two CAMs differ. CAM I has an inject depth of about 50 to 70 mm, 28 days compressive strength of about 1.8 MPa, and elastic modulus of 100 to 300 MPa; concerning CAM II, on the other hand, the inject depth is about 20 to 40 mm, 28 days compressive strength is about 15 MPa, and elastic modulus is about 7000 to 10,000 MPa. Previous researches have demonstrated that CA mortar possesses viscoelastic properties [16,17,18], and therefore, their performance properties and behaviors are subordinate to pressure, temperature [19], and strain rate. The viscoelastic properties of CAM are the determining factors of its structural functionalities. Moreover, with the properties of asphalt being more influenced by temperature as compared to cement, the dynamic mechanical response of CA mortar under varied service temperatures may differ [11]. Both CAM I and CAM II have their advantages and preferences. Engineers consider the available data and decide on the one to be used for certain track construction. For instance, more asphalt (higher A/C) is required in CAM I when the railway structure requires more damping performance; whereas more cement (lower A/C) is included in CAM II if the railway structure needs more strength compared to the damping performance [20].

The interaction between the constituent materials of CA mortar influences its properties; the interactions between components of CA mortar include the impact of cement on the breakdown of emulsified asphalt and the effect of emulsified asphalt on the hydration process of cement [21,22]. Water coming from the demulsification process of emulsified asphalt triggers the hydration process of cement. Thus, this provides solutions to the inconsistencies between water repelling during the demulsification of asphalt emulsion and water demand of the cement hydration process [20]. Therefore, CA mortar does not combine only the advantages of cement and emulsified asphalt materials but also improves the shortcomings of the two materials. Properties of CA mortar such as the damping performance and strength characteristics are greatly affected by the amount of asphalt emulsion and cement. The damping performance and strength characteristics of CA mortar are determined by various parameters by engineers during design, which includes the type of railway track structure, load, environmental conditions, etc.

The recent research studies concerning CA mortar have prioritized its mechanical properties and the effects of train loading and environmental conditions (such as temperature and moisture) on the long-term performance of CA mortar. Currently, the incorporation of industrial by-products, naturally occurring minerals, and other supplementary cementitious materials (SCM) as a partial replacement of cement in the production of CA mortar has also been getting attention from researchers across the globe. This technique has contributed to ensuring a sustainable environment and reducing the cost of production of CA mortar. Recent researches have indicated the potentials of CA mortar to be applied in ballasted tracks, but unlike the ballastless slab track, in a ballasted track, CA mortar is not a layer or structural member on its own, but rather it is incorporated or mixed with the ballast to form a new composite material named CA mortar-stabilized ballast. The new material is anticipated to achieve the merits of the ballastless track as well as be used as a fouled ballast rehabilitation solution, and hence reinforce the ballast layer by improving its durability and also reduce the construction time [14,23]. In related research, Le et al. [23] proposed a technique that assesses the plausibility of stabilizing the ballast using CA mortar, as an eminent solution to minimize losing the track quality as a result of particle abasement and ballast settlement. Based on the technique they proposed, pouring fresh CA mortar on the ballast layer will cause the CA mortar mixture to flow through the ballast system, thereby coating the ballast particles and creating strong inter-particle bonding [24]. The bonded material will be able to achieve stiffness from cement hydrates, and viscoelasticity from the asphalt membrane [24]. Although the properties of asphalt material are delicate to climatic conditions such as moisture and temperature, in this technique, the incorporation of cement will mitigate this problem. Though this technique receives attention from researchers all over the globe, however, the life-cycle cost analysis has been limited in this regard.

Although studies on the performance properties and structure of CA mortar have been previously reported, a detailed study on the composition mechanism and compatibility among constituent materials is rarely reported. These aspects have great influences on the properties of CA mortar. Hence, a comprehensive review on the compatibility between constituent materials, the microstructure of cement asphalt binder (CAB), structural development, and the effect of A/C on properties of CA mortar is presented in this study. The paper reviews essential research outcomes related to CA mortar composites, properties, and the compatibility among constituent materials. Based on previous researches, the current review summarizes the interaction among asphalt emulsion and cement and their influences on the performance properties of CA mortar. Basic information regarding the microstructure of cement emulsified asphalt binder (CAEB), A/C ratio, and the use of admixtures as a partial substitution of cement in the preparation of CA mortar are discussed and presented comprehensibly. Eventually, possible arrears for future research are suggested based on previous studies reported.

## 2. Preparation of CA Mortar

CA mortar is prepared using an appropriate mix proportion of constituent materials and depending on the type of CA mortar and where it will be used or applied, the proportion of materials varies. Mixture proportions are normally selected following the standards set by the relevant regulatory bodies in different countries. These proportions include the percentage by mass or volume of each of the constituent materials, as well as the various ratios such as A/C, ratios of sand to cement (S/C), water to cement (W/C), etc. According to the chosen mix proportions, the materials are then mixed using the designated method and apparatus. Consequently, in the construction of a non-ballasted plate track, CA mortar is grouted within the space between the bottom concrete trackbed and the top track slab with its gravitational force; thus, it needs to possess good properties to alter to such a technique [3,25,26], and to enhance the stability and comfort degree of the ballastless slab track structure [27].

### 2.1. Mixing Method

**Figure 3:**
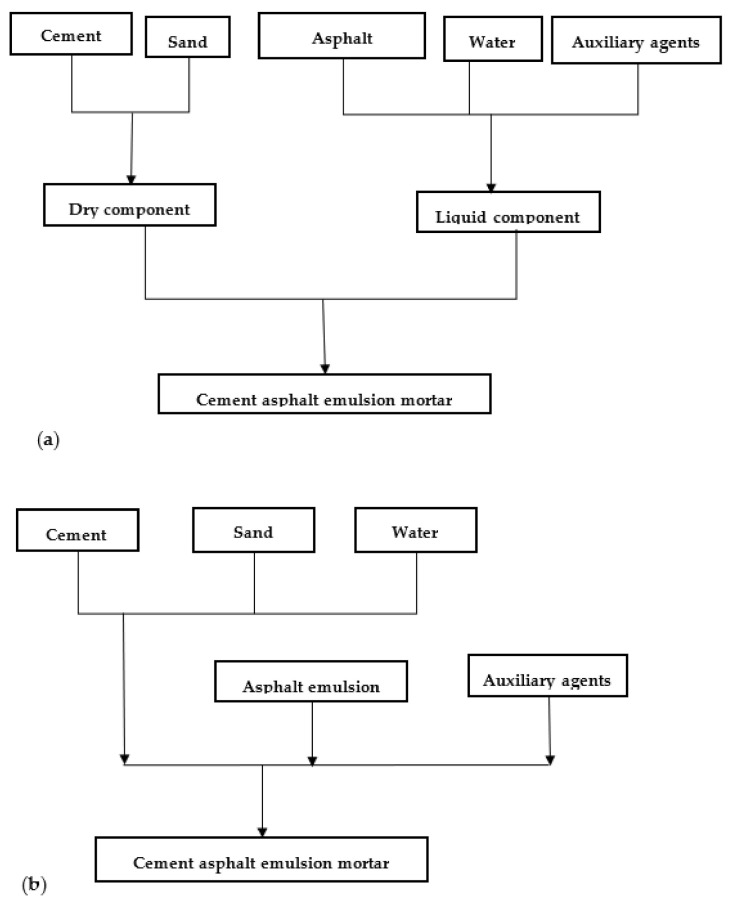
Flow chart for CA mortar preparation; (**a**) dry mixing method (**b**) wet mixing method.

There are generally two mixing methods commonly used for the laboratory preparation of CA mortar; these methods are known as dry and wet mixing methods. The major contrast between the two methods as illustrated in Figure 3 is that liquid components (asphalt emulsion, water, and auxiliary agents) and dry components (cement and sand) are mixed independently in the dry-mixing method, and after which they are blended. Contrarily, for the wet-mixing method, firstly, cement and sand are mixed with water and superplasticizer separately, and then the emulsified asphalt and other auxiliary agents are added [14]. The mixing time and mixing speed (critical mixing speed) are selected appropriately following the relevant standards. It was reported that mixing time, mixing speed, and the sequence of adding raw materials affect the properties of CA mortar [28,29]. In CA mortar production, the mixing process should be aimed to achieve two major functions: one is the dispersion of particles on the macroscale and microscale, and the other is the entrainment of air bubbles. To produce CA mortar with desirable mechanical properties, the air content should be within the range of 8% to 12%; and it was reported that mixing speed, mixing time, and fluidity had direct effects on the air content of CAM [28]. The mixing speed and defoaming agents influenced air content by affecting air bubble entrainment and retention. The air content in CA mortar is a result of air entrainment and air retention, these two phenomena are related to the mixing process and the properties of fresh or liquid CA mortar (i.e., density, viscosity, and surface tension). Defoamers or defoaming agents are used in CA mortar preparation to control the air content together with the appropriate mixing speed and mixing time. Defoamers lower the surface tension of the air bubbles; they make the air bubbles less stable, and thus, they can easily burst or rupture. Additionally, deforming agents form an elastic film on the paste surface and consequently prevent the formation of air bubbles. Concerning mixing speed, when the mixing speed is high, the defoamers also try to depress the air-entraining ability; but when the mixing speed is low, the defoamers may have a slight influence on air-entraining ability [28]. Therefore, a critical mixing speed should be maintained. Anything below or above critical speed will have a direct influence on the air content of CA mortar.

### 2.2. Structural Development of CA Mortar

When cement and asphalt emulsion form a mixture, cement hydration and the demulsification of emulsified asphalt begin to take place; the asphalt droplets usually form asphalt film after demulsification and cement grains are hydrated out–in, which consumes the water and increases the solid contents [3]. The structural development of CA mortar is associated with the change of state of the mixture of cement and asphalt emulsion from flow paste to a solid or hardened mortar. This mixture changes from flow or liquid state to plastic state and then to the solid-state; this process can be divided into four major phases or states: the dispersion state, the phase of interaction between emulsified asphalt with cement, the asphalt film structural formation phase, and structural development of solid mortar [3].

#### 2.2.1. Dispersion State

**Figure 4:**
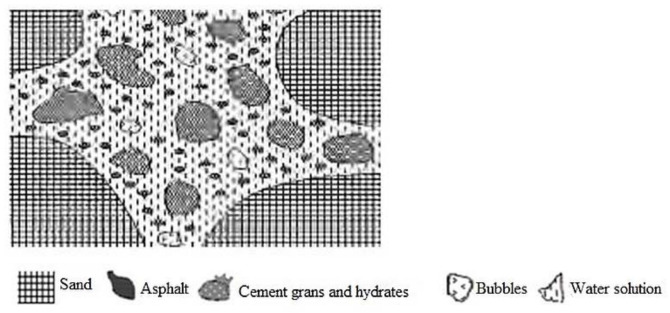
Dispersion state [3].

In the dispersion state, cement particles and asphalt emulsion droplets disperse independently within the fresh CA paste [3]. Figure 4 illustrates the dispersion state in the structural development of CA mortar.

#### 2.2.2. Interaction between Cement and Asphalt Emulsion

**Figure 5:**
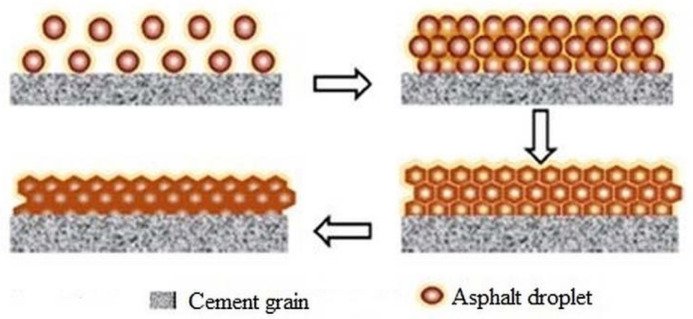
Schematic representation of asphalt net formation on cement grain [31].

The interaction among the components of CA mortar influences its behavior and properties. Cement and emulsified asphalt are the two major components in CA mortar, comprising about 21% and 25% of the whole volume of CA mortar, respectively [30]. When asphalt emulsion and cement are mixed, they interact with each other in such a way that the consumption of water due to the hydration of cement accelerates the breakdown of emulsified asphalt; likewise, the adsorption of asphalt droplets on the surface of cement grains slows down the cement hydration process [1,31,32]. In the process of the hydration of cement, several ions such as K⁺, Na⁺, Ca^2^⁺, SO₄^2−^, and OH^−^ are discharged at varied concentrations into the pore solution [33]. Ca(OH)₂ from the hydration of cement neutralizes the acid in emulsified asphalt, as such, hydration product begins to form when the Ca(OH)₂ concentration is saturated. At the same time, an asphalt emulsion is absorbed onto the cement grains and cement hydration products [3]. The interaction among cement and emulsified asphalt are usually represented experimentally by two aspects: the acceleration of asphalt emulsion breakdown due to the process of hydration of cement, and the retarding effect on cement hydration caused by the adsorption of asphalt droplets on the surface of cement grains [1], as illustrated in Figure 5.

In the related literature, Jing et al. [30] reported that emulsified asphalt affects both the rate of cement hydration and the degree of hydration, stating that emulsified asphalt retarded the rate of the hydration of cement but did not change the composition of cement hydrates. The degree to which asphalt emulsion affects the hydration process relies on the type or class of emulsified asphalt used. Nevertheless, the incorporation of anionic emulsified asphalt into cement causes a remarkable delaying effect than cationic emulsified asphalt [31]. This could be due to the different manner of their adsorption to the surface of cement grains. Conversely, it was reported in another research that water loss by emulsified asphalt to the cement hydration process remarkably affects the stability of both anionic and cationic emulsions, as well as accelerating the flocculation speed of both emulsions [33]. Most studies suggested that the interaction between asphalt emulsion and cement is mostly physical, as no chemical reaction between the two was reported. According to a study conducted by Yang et al. [34], when emulsified asphalt and cement are blended, no chemical reaction could be detected except for the cement hydration process.

#### 2.2.3. Formation of Asphalt Net Structure

**Figure 6:**
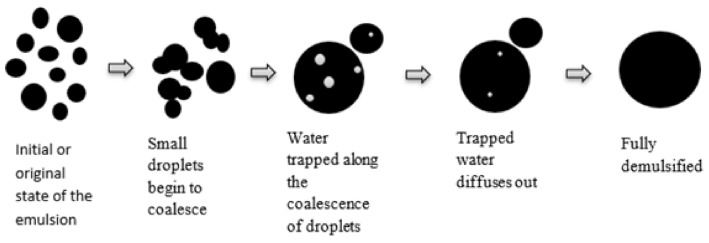
Stages in the breakdown or demulsification of asphalt emulsion [37].

Once in contact with water, Portland cement begins to hydrate, set, and then hardens due to the number of chemical reactions among different chemical substances and water [35]. Emulsified asphalt, on the other hand, is formed by the process of dispersing small beads of asphalt or bitumen in water. Emulsified asphalt can be solidified or demulsified by flocculation and coalescence of asphalt droplets [1,36], as illustrated in Figure 6.

As soon as cement and emulsified asphalt are mixed, cement consumes water from emulsified asphalt, and this consequently decreases the distance between asphalt droplets. As a result, asphalt droplets make contact and coalesce and finally form a continuous asphalt film, as illustrated in Figure 5.

#### 2.2.4. Structure and Strength Development of Hardening Mortar

The mechanical properties of CA mortar are closely related to its composition [38]. The consumption of water by cement promotes the breakdown of emulsified asphalt, and the continuous hydration process brings about the rapid consumption of free water from emulsified asphalt and the absorption of asphalt droplets on the surface of cement grains. Consequently, cement hydrates are formed. Meanwhile, asphalt particles coalesce together to form a membrane around the surface of hydration products, fine aggregates, and non-hydrated cement particles. Further cement hydration causes the continuous breaking of asphalt particles, thereby forming an asphalt net film. At last, the asphalt membrane gets stuck to the surface of the hydration products and forms a spatial network structure, and then fine aggregates and cement hydration products fill the asphalt net structure as a filler [3,18,39]. Asphalt droplets split gradually, and its membrane sticks to cement hydrates (as depicted in Figure 5) to try and stop further cement hydration. A study shows that hydration products usually impale the asphalt membrane for further hydration to take place [39]. The strength and density of CA mortar usually increase with time; this is probably due to the slow down effect of the asphalt membrane on the contact of cement grains and water [3].

The strength development mechanism in CA mortar relies highly on the A/C ratio, for the two types of CA mortar (i.e., CAM-I and CAM-II), the strength formation mechanism differs. For CAM-I with a high A/C ratio, the asphalt phase usually dominates the structure of the hardened CA mortar and therefore weakens the framework of the hydration product of cement. Meanwhile, the asphalt network is also weak; hence, the whole structure of CAM-I experiences weak strength, especially at an early age. For CAM-II, the asphalt to cement ratio is low, the framework formed by hardened cement paste dominates the structure of the hardened CA mortar and therefore determines its strength [39]. The higher the cement content, the faster the strength of CA mortar at an early age; this indicates that the A/C ratio significantly affects the early strength gain by CA mortar. Qiang et al. [39] studied the strength mechanism of CA mortar prepared with different A/C. The results of their study indicated that emulsified asphalt slows down early cement hydration, and the asphalt membrane negatively influenced further hydration of cement. They reported that for CAM-II, which possesses a lower A/C and a high elastic modulus, hardened cement paste is the essential structural skeleton that influences strength development. While for CAM-I, which has a higher A/C and low elastic modulus, the framework formed by the asphalt film membrane and the framework formed by hardened cement paste are responsible for the strength development [39]. Their study clearly shows that the A/C ratio plays a vital role in determining the properties of CA mortar, but no detailed explanation was found in their study as to the effect or contribution of using different types or classes of emulsified asphalt on the strength development of fresh and hardened CA mortar.

### 2.3. The Microstructure of CA Mortar

The fine structure of a material as revealed by microscopy is the microstructure of that material. The microstructure of CA mortar has been used by researchers to study or examine an experimental result that has to do with the properties of CA mortar. Scanning electron microscopy (SEM) image analysis and mercury intrusion porosimetry (MIP) are among the techniques commonly used to study the microstructure of CA mortar. The microstructure of CA mortar reveals the fine structure of cement hydration products bind together with the asphalt membrane structure, the combination of which is referred to as cement asphalt binder (CAB). Cement asphalt binder is characterized by a two-phase system, including the asphalt binder phase and the hardened cement paste (hcp), which are produced from the demulsification of asphalt emulsion and cement hydration, respectively [1].

When cement and emulsified asphalt are mixed, cement consumes water, and cement hydrates such as calcium silicate hydrate (C-S-H), calcium hydroxide (CH), and ettringite are produced. This will result in the formation of a binding phase made up of hcp. C-S-H gel is the major component of hcp, therefore, accountable for the binding and strength development [1]. Furthermore, at the end of the cement hydration, the porous C-S-H gel takes up a large volume of hardened cement paste and forms a compound microstructure [1]. With regards to asphalt binder, being an organic material, asphalt binder has a complex microstructure which is decided by its chemical components; asphalt binder is generally regarded as having a colloidal system, and therefore, at a macroscale level it can be regarded as one continuous phase [1,40,41].

**Figure 7:**
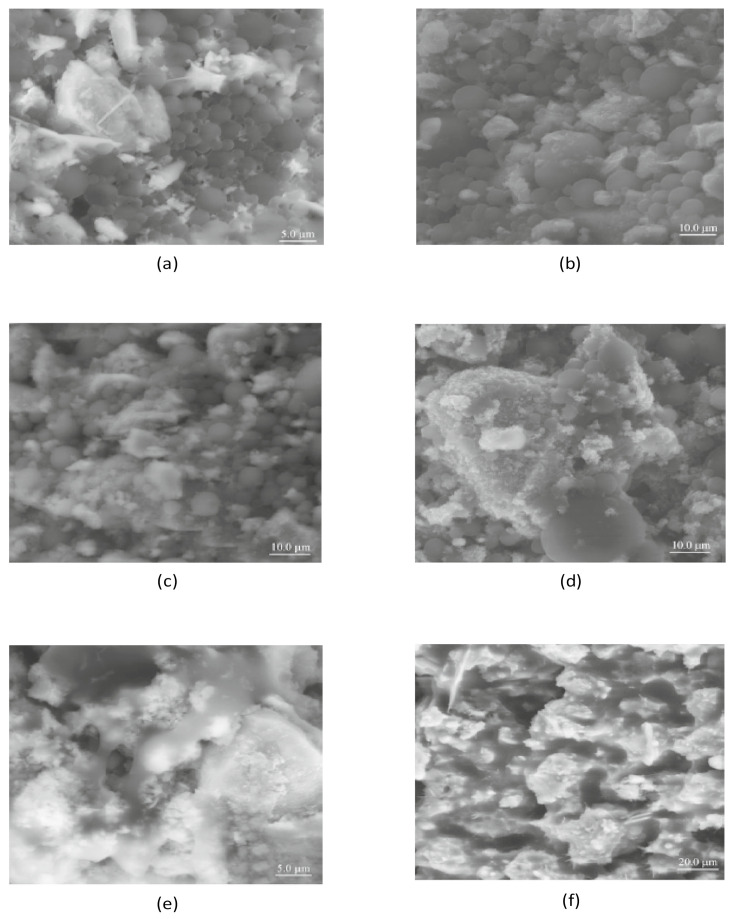
SEM images for the microstructure of CA paste hydrating at (**a**) 0 h after mixing (**b**) 3 h (**c**) 6 h (**d**) 12 h (**e**) 24 h (**f**) 28 days curing age [42].

The microstructure of the CAB system is highly controlled by the content of asphalt binder and hcp, that is to say, A/C. With respect to A/C, asphalt binder and hcp each compete to be the dominant binding material in CAB. Yongliang et al. [17] reported that the stress and elastic modulus of CAMs decrease with an increase in A/C and temperature. In addition, they also noted that CAM with a higher A/C is more sensitive to temperature and loading rate compared to CAM with a lower A/C. This is to say, with the increase in temperature and or A/C, the visco-elasticity tends to be more significant [17]. This is because hcp is brittle with high elastic modulus and compressive strength whose properties are greatly influenced by curing age, while asphalt, on the other hand, is a visco-elastic binder at room temperature with a low modulus. Increasing asphalt content will change the microstructure of the CAB and consequently affect the mechanical properties of CA mortar. Moreover, due to the nature of the asphalt binder, the coordinate perception of the microstructure of a hardened CAB is not so straightforward. Asphalt binder is soft at encompassing temperature and very sensitive to environmental temperature; this implies that the morphology of CAB can be effortlessly altered during the sample preparation process and the operation of the microscope. In the related literature, using a scanning electron microscope under environmental conditions (ESEM), Wang et al. [42] studied the microstructure of fresh CA mortar prepared with an A/C and W/C of 0.3 and 0.4, respectively. The microstructure of the CA mortar sample at different curing ages of 0, 3, 6, 12, and 24 h after mixing was studied. Figure 7a–e displayed the ESEM photograph of fresh cement–asphalt paste according to their results. At 0 h, that is just after mixing, Figure 7a shows that asphalt particles blended uniformly with cement particles with no interaction. At 3 h, some cement hydrates could be seen (Figure 7b). At 6 h after mixing, some cement hydrates could also be observed but in a limited amount, as shown in Figure 7c; this indicated that cement hydration is in the acceleration period, so many products are produced at 6 h after mixing [42]. At 12 h after mixing as shown in Figure 7d, a significant amount of cement hydrates had formed, and also the C-S-H layer could be identified. At 24 h after mixing, as depicted in Figure 7e, a large amount of cement hydration products with ahigh consumption of free water resulted in emulsion splitting that consequently resulted in the formation of the asphalt membrane. When the demulsification of emulsified asphalt was completed, the asphalt membrane gets attached to the surface of cement hydrates. Moreover, it could also be observed in Figure 7e that, not all hydration products were entirely enclosed by the asphalt membrane; this is because some portion of un-hydrated cement might still be in contact with water for further hydration. Thus, the hydration product of cement contributes to the elasticity of the CA mortar, while the asphalt membrane contributes to the viscosity of the CA mortar [42]. Additionally, using SEM, they conducted a similar investigation on the microstructure of hardened cement-asphalt paste (CA paste) prepared using A/C of 0.30 and W/C of 0.50 when the curing age reached 28 days. It can be observed in Figure 7f that at 28 days, a permeating network of cement hydration products and asphalt membrane had been formed [42]. It can also be observed that the dominant phase for this structural framework is hcp; this is as a result of a lower A/C that indicated a high content of cement compared to asphalt; this structure decides the strength of CA mortar. Thus, their results reaffirmed that A/C greatly influences the compressive strength of CA mortar. Nevertheless, it ought to be noted that, in most of these studies some preliminary efforts were all reasonably made in clarifying the complex microstructure of CABs. An advanced research study is still required to understand more about the microstructure of CABs, especially for longer curing age and that of CA mortar at some time after it is exposed to the service. This will help in understanding the behavior and adherence of CA mortar toward repair materials. In addition, a broad understanding of the microstructure of CABs is vital as the properties and behavior of CABs determine the properties and behavior of CA mortar.

### 2.4. Damping Performance of CA Mortar

The damping performance of a material refers to the dissipation ability of vibration energy in that material. The damping property of a system is also the capability of that system to transform vibrational energy into other forms of energy [43,44,45]. Strength and damping ability are among the basic functions for which CA mortar is designed to serve in the structure of a non-ballasted track system. The basic concept of damping is the loss of energy and lots of innovations related to damping concentrate on converting the induced vibrational energy to other forms of energy so that a system can return to its initial state promptly [46,47]. The interaction between cement and emulsified asphalt ensures that after the process of cement hydration, cement hydrates provide strength to the CA mortar while emulsified asphalt provides toughness to the CA mortar after demulsification. Thus, the composition of cement and asphalt emulsion produces a material (CA mortar) with adequate damping ability [11,17,48]. The damping performance and strength of CA mortar are affected mainly by the A/C ratio. Previous researches have shown that the damping performance of CA mortar increases with an increase in the A/C and temperature [11,20,49]. In the study of Leiben et al. [20], it was reported that the increase in the A/C improves the ability of CA mortar to resist deformation and also enhances its plasticity; increasing the A/C brings about an increase in the amount of asphalt, which significantly increases the amount of asphalt film that wraps the surface of cement hydrates; this consequently improves the damping ability of CA mortar. Therefore, the appropriate A/C is needed to synchronize the damping performance and strength characteristics for various applications of CA mortar. Another research was conducted by Li et al. [50] concerning the dynamic properties of CA mortar modified with rubber powder (RP). They analyzed the vibration frequency and damping ability of a sample specimen (a beam) of CA mortar through free attenuation vibration tests. Their studies indicated that there was no remarkable change in the initial frequencies for CA mortar incorporated with rubber powder; however, there was an increase in higher frequencies and the damping ratio of the sample specimen when the amount of rubber powder was increased. Therefore, the incorporation of rubber powder into the CA mortar can enhance its damping ability for good energy absorption and vibration dissipation. Similar conclusions have been made in the related literature [51], where it was documented that the incorporation of rubber powder or fiber can ameliorate the damping ability of cement mortar; nevertheless, the incorporation of fiber was found to be more efficient than RP in enhancing the damping ratio of cement mortar [51].

## 3. Compatibility between Major Constituent Materials of CA Mortar

In CA mortar preparation, the mixture of its constituents is generally associated with two major processes, i.e., the cement hydration process, and the breaking down of emulsified asphalt; these processes affect each other. Therefore, cement and emulsified asphalt should have an appropriate ratio in which the rate of hydration of cement will match the rate of demulsification of asphalt emulsion [3]. The compatibility between cement and emulsified asphalt is termed as having an appropriate proportion of cement and emulsified asphalt in which the cement hydration rate matches the emulsion breaking speed. CA mortar is a profoundly flowable grout made with different heterogeneous constituents of distinctive proportions, and thus, the compatibility among them is very imperative as they impact both fresh and hardened properties of CA mortar. The setting process of CAB has major significance on the properties of CA mortar, and as such, an appropriate A/C is required for this process together with a compatible rate between the demulsification of asphalt emulsion and cement hydration, which is imperative to the homogeneity of CA mortar [3,52].

### 3.1. Significant of Cement on Demulsification of Asphalt Emulsion in CA Mortar

Water loss is induced in emulsified asphalt due to the fact that the hydration of cement triggers the demulsification of emulsified asphalt, which consequently affects the chemical stability of asphalt emulsion. Besides, compatibility between emulsified asphalt and cement could be estimated using the chemical stability of emulsified asphalt when mixed with cement [33]. The hydration of cement affects the chemical stability of emulsified asphalt in different ways; the loss of water to cement hydration causes the spaces between the asphalt particles within the emulsion to collapse, and this increases the chances of the coalescence of asphalt micelles. Additionally, the alkalinity of cement neutralizes the acid in cationic emulsion that results in the rise of the pH value of cationic emulsion. Wang et al. [33] studied the effect of pH value, Ca^2^⁺ concentration, and water loss due to hydration of cement on the chemical stability of asphalt emulsion. Their results indicated that the chemical stability of the cationic asphalt emulsion is highly influenced, as it suffered a rise in the pH value. They reported that the addition of an anionic emulsifier counters this effect as it enhances the emulsion’s resistance to alkaline attack. For anionic emulsion, on the other hand, its chemical stability was slightly improved by the rise in pH value. The effect of Ca^2^⁺ on the chemical stability of anionic and cationic asphalt emulsions was modest. The loss of water by asphalt emulsion due to the hydration of cement has a major influence on the chemical stability of both anionic and cationic emulsions, as it speeds up the flocculate speed of both emulsions. In the study of Hu et al. [32], the adsorption manners among emulsified asphalt and cement in CA mortar were studied, and it was reported that cement hydration speed up the demulsification of asphalt emulsion, which in return accelerated the adsorption of asphalt droplets onto the surface of cement grains, thus, increasing the particle size in the cement–asphalt emulsion (CAE) system. They used the filtration method to study the adsorption property of asphalt particles to the surface of cement grains, while the influence of adsorption behavior of asphalt droplets in the emulsion on the particle size of the CAE system was evaluated using a laser particle size analyzer. Their results indicated that the adsorption ratio corresponded with the change in particle size, implying that particle size increase in the CAE system was mostly connected with the adsorption behavior. Cement was proved to be an effective adhesive agent for asphalt emulsion mixtures [53]. Most of the researchers have studied the influence of cement on the chemical stability of asphalt emulsion, however, studies on how the influence of cement on emulsified asphalt affects the long-term performance and strength characteristics have rarely been considered.

### 3.2. Influence of Asphalt Emulsion on Cement Hydration in CA Mortar

**Figure 8:**
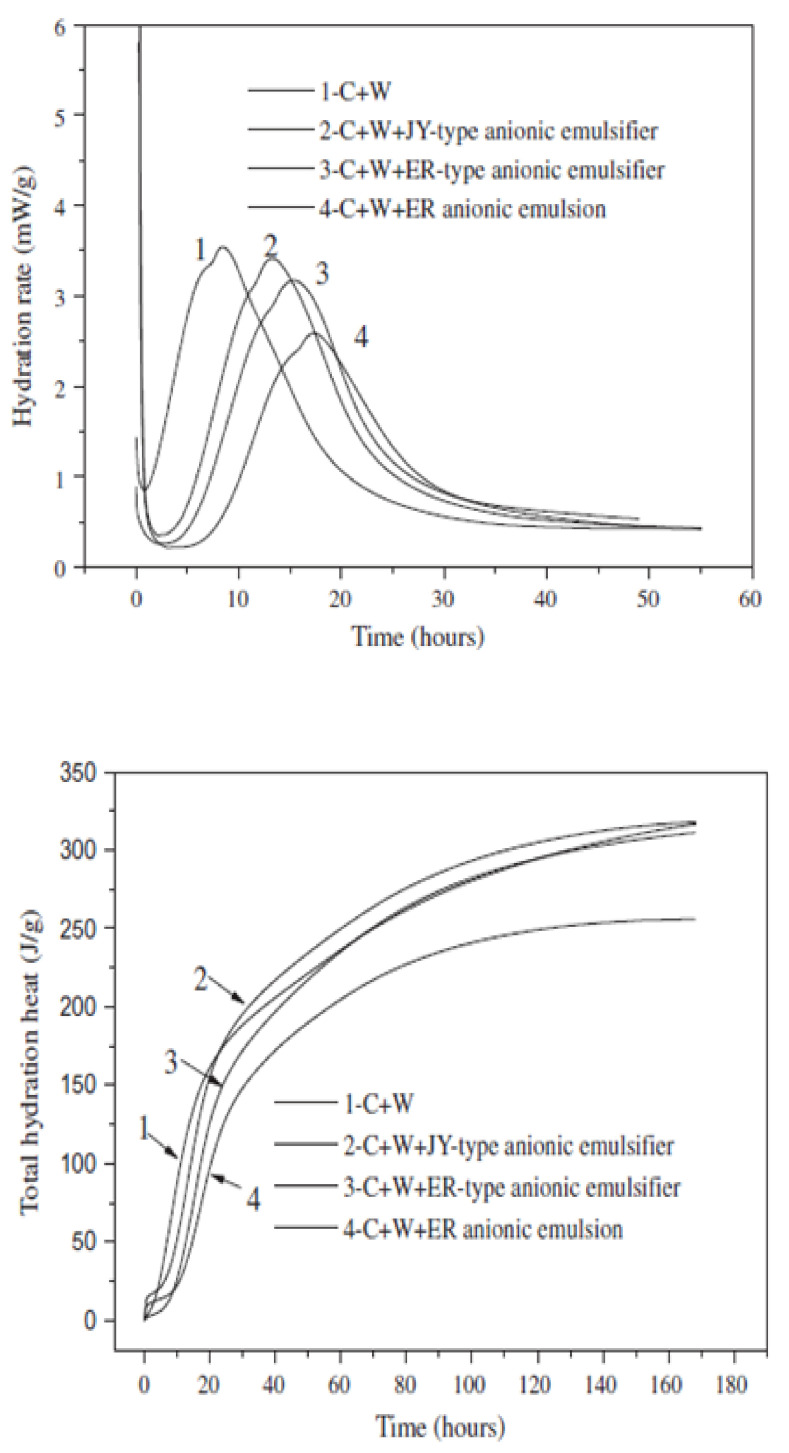
Isothermal calorimetry curves for cement with anionic emulsion and anionic emulsifier [55].

**Figure 9:**
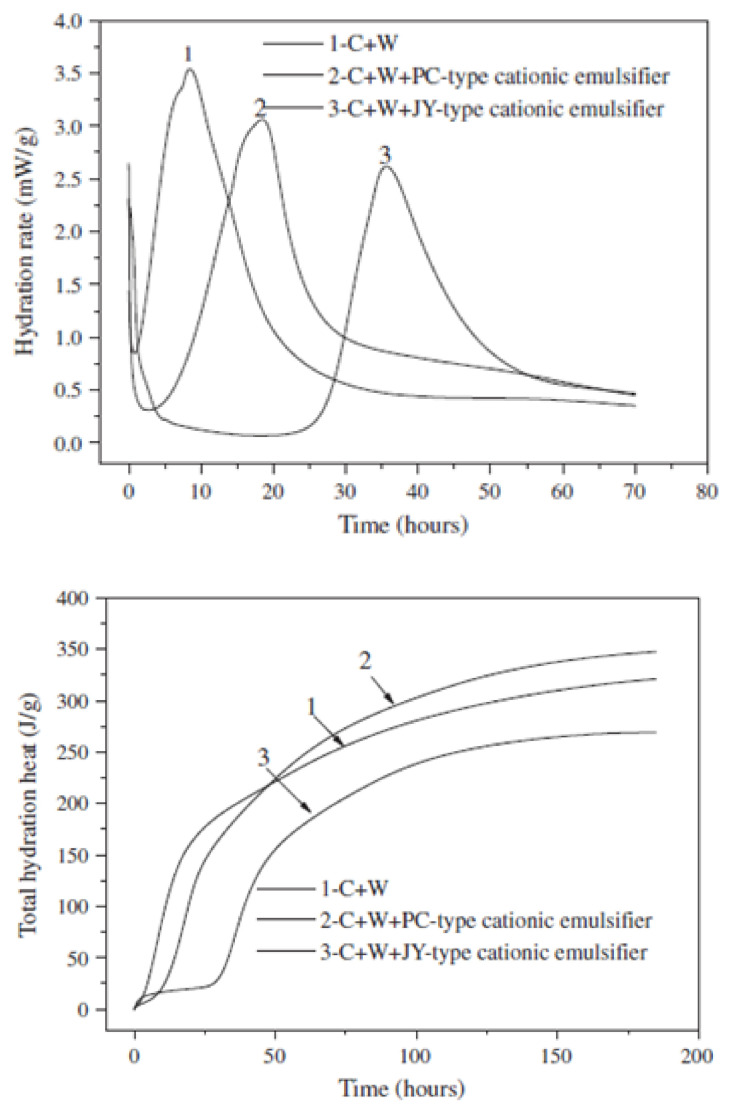
Isothermal calorimetry curves for cement with cationic emulsifiers [55].

In the mixture of asphalt emulsion and cement, asphalt emulsion affects the cement hydration process; water released from the breaking of asphalt emulsion and the water added to the mixture reacts with the cement to facilitate the cement hydration process. According to Qiang et al. [39], the cement hydration process can be divided into five stages; the rapid heat release period, dormant period, acceleration period, deceleration period, and steady period. In the process of cement hydration, a certain concentration of calcium and hydroxide ions needs to be achieved before the formation of crystal nuclei; this is what brings about the dormant period. Besides, the dormant period normally appears during the initial hydration of CAB, and it can be prolonged with the increase in the A/C due to the effect of asphalt and emulsifier [39]. A study conducted by Jing et al. [30] compares CA mortars prepared with two different asphalt emulsions with cement mortar in terms of the hydration process of cement, microstructure, compressive strength, and the chemical composition of cement hydrates. Their studies indicated that with the addition of asphalt emulsion, exothermic rate, temperature, and rate of hydration were lowered in CA mortar than in cement mortar. Additionally, their energy dispersion (EDS) and SEM analysis showed that asphalt emulsion retarded the rate of cement hydration but did not alter the chemical composition of cement hydrates. Moreover, the slow down effect increased when the A/C was increased [31,32,34,39,54]. In the work of Tan et al. [55], the slow down effect of emulsifiers on cement hydration was evaluated by measuring the setting time of cement, cement hydration rate, and X-ray diffraction analysis. Two anionic emulsifiers (ER and JY anionic emulsifiers) and two different types of cationic emulsifiers (PC and JY cationic emulsifiers) were used to prepare cement–emulsifier paste samples following the mix proportion given in Table 1; the setting time of cement with different types of emulsifier was measured at a test temperature of 20 ± 0.02 °C. The hydration heat for the samples was also measured by the isothermal calorimeter and the results are compared with the hydration heat of plain cement paste as presented in Figure 8 and Figure 9. Based on their results, they concluded that emulsifier has a cogent slow down effect on the hydration of cement which is connected to the type and dosage of emulsifier and also to the ratio of emulsifier to cement. A momentous difference in cement hydration rate, setting time, Ca(OH)₂ content, and hydration heat was observed for samples with different emulsifiers. Besides, emulsifiers with a high slow down effect on the hydration of cement cause a loss in the hydration heat, cement hydrates, and also aborts hydration at an early age. Thus, the appropriate dosage of emulsifier with the less slow down effect is recommended to be used in the preparation of asphalt emulsion for CA mortar [55].

In another research work, Wang et al. [56] evaluated the hydration process of cement and viscoelastic properties in two different types of CA mortar prepared with different types of asphalt emulsion. They reported that the incorporation of emulsified asphalt remarkably influenced the hydration of cement, and the total heat of hydration was found to decrease with an increase in the A/C. Furthermore, they also reported that the slow down effect on cement hydration due to anionic emulsion was more than that of the cationic emulsion—this was also reported in the related literature [31]—thus, anionic emulsion resulted in a lower hydration heat than cationic emulsion. On the contrary, CA mortar made with cationic emulsion manifested a high compressive strength when compared to CA mortar made with anionic emulsion. Concerning workability, anionic emulsion enhanced the workability of CA mortar more than cationic emulsion [56]. This may be connected to the manner of their adsorption on the surface of cement grains, and since anionic emulsion retards the cement hydration process, the setting time of cement will also be prolonged; this helps in improving the workability of CA mortar made with anionic emulsion.

### 3.3. Effect of Asphalt to Cement Ratio (A/C) on the Mechanical Properties of CA Mortar

The A/C ratio is one of the most important parameters that determine the properties of the CA mortar. A higher A/C (usually above 0.6) signifies that the amount of asphalt in the CA mortar is more than the amount of cement, and therefore the properties of CA mortar made with a higher A/C are dictated by asphalt. Likewise, a lower A/C (below 0.6) means that the content of cement exceeded the content asphalt in CA mortar, thus, the behaviors of CA mortar made with a lower A/C are controlled by cement. Cement and asphalt are entirely different materials in CA mortar, and although there are other constituent materials in CA mortar, the A/C is the major factor that controls the strength and modulus of elasticity in CA mortar compared to the W/C and the ratio of sand to cement (S/C) [39,57]. Both emulsified asphalt and cement contribute to the mechanical properties of CA mortar. When introduced or incorporated into cement mortar, emulsified asphalt generally has a negative influence on its strength and modulus of elasticity but improves its deforming ability [10,39,58,59]. Studies show that CA mortar with a higher A/C is associated with low strength and higher damping ability, while CA mortar with a lower A/C is associated with a higher strength but low damping ability. Although there are various A/Cs used in CA mortar preparation, in China’s ballastless slab track, the most commonly used A/C and W/C for CA mortar in practical application are 0.85 and 0.68, respectively [48]. It was reported in related research works that the decreasing rate of elastic modulus and compressive strength of CA mortar decreases when the A/C is increased, and properties of CA mortar possessing a higher compressive strength and elastic modulus have utmost sensitivity to the A/C than those of CA mortar with alow compressive strength and elastic modulus [39,58]. Previous researches have shown that the properties of fresh and hardened CA mortar are mostly dependent on the A/C, but the relationship between the two is nonlinear [60]. Fang et al. [57] further investigated the influence of mix parameters such as the A/C, W/C, and S/C on the dynamic mechanical properties of CA mortar. The results of their studies indicated that storage modulus (E’) increases with a decrease in the A/C and W/C ratios and increases with an increase in the S/C ratio. This is related to the increase in cement content and cement hydrates that is associated with a decrease in the A/C; cement possesses a higher elastic modulus than asphalt, so increasing cement content causes an increase in the elastic modulus of CA mortar; this is in line with some other results found in the related literature [59]. With regard to the W/C, increasing the W/C results in higher capillary porosity which leads to the decrease in the volume fraction of solid phases of CA mortar composites (i.e., cement hydrates, sand, and asphalt); and Young’s modulus of the material decreases with the increase in porosity [61]. Thus, increasing the W/C ratio leads to a decrease in the storage modulus. Increasing the S/C on the other hand means more sand is added to the mixture of CA mortar, which results in an increased volume fraction of its component, and sand possesses a higher Young’s elastic modulus than that of asphalt and cement. Therefore, increasing sand content leads to an increase in the elastic modulus of the CA mortar [57]. Additionally, based on their results, the damping performance of CA mortar characterized by (tan ⸹) increases with an increasing W/C and A/C. This is because the asphalt content of CA mortar is responsible for its damping behavior. Hence, increasing the A/C increases the amount of asphalt, which consequently increases the damping performance of the CA mortar. Additionally, CA mortar with a lower W/C ratio has higher asphalt content and low porosity than CA mortar with a higher W/C. Thus, a higher W/C is associated with high porosity in CA mortar; therefore, the presence of porosity contributes to the increase in the damping capacity due to an increase in W/C. According to their results, damping ability (⸹) shows slight dependence with the S/C, especially for CA mortar with a low A/C, but for CA mortar with a high A/C, ⸹ slightly decreased when the S/C is increased. This is because the addition of more sand weakens the concentration of asphalt; hence, ⸹ slightly decreases when the S/C increases [57]. Ultimately, they established a relationship between E’, ⸹, and volume fraction of phases in CA mortar as shown in the following equations [57];

(1) $$E\prime \;=\;a_{1}V_{a}^{3}\;+\;a_{2}V_{c}^{3}\;+\;a_{3}V_{s}^{3}\;+\;C$$

where *E′* is the storage modulus; *Vₐ*, *V_c_*, *Vₛ* is the volume fraction of asphalt, cement hydrates, and sand, respectively: *a₁*, *a₂*, *a₃* and C is the temperature-dependent factors (with the same form of a = K₁ + Tb₁, where K₁ and b₁ are constant; and T is the temperature (°C)) [57].

(2) $$\tan\;⸹\;=\;\frac{AV_{a}}{1+B\left(1-V_{a}\right)}$$

where tan ⸹ is the damping ability of CA mortar; $V_{a}$ is the volume fraction of the asphalt phase; *A* is the parameter related to the damping of asphalt; and *B* is the factor accounting for the interaction between asphalt and inorganic phases (i.e., cement and sand), respectively. *A* and *B* are temperature dependent, and therefore, they are constant at different temperatures [57].

They demonstrated that the proposed relationship in Equation (1) can be used to forecast the E’ of various CA mortars at various temperatures, while Equation (2) gives the relationship between the damping ability and the amount of asphalt in CA mortar. The calculated data from the proposed equations were consistent with their experimental data. Nevertheless, the limitation of the established model, especially Equation (1), is that it did not clearly show the relationship between the A/C and the elastic modulus of CA mortar, and the W/C was not clearly captured in Equation (2), these parameters (A/C and W/C) significantly affect damping performance and elastic modulus of CA mortar, respectively.

In related research work, Ouyang et al. [60] further investigated the elastic modulus and compressive strength of CA mortar with different A/Cs and temperatures. Their experimental results also indicated that there is a decrease in the compressive strength of CA mortar with an increase in the asphalt to cement volume ratio (*V_A_/V_C_*); the elastic modulus also shows a similar trend as that of compressive strength. This is obvious, as explained in the previous literature, that with an increase in the A/C, cement content is reduced, and this results in a reduction of cement hydrates that are in charge of the strength of CA mortar, [59]. Additionally, increasing the A/C increases the content of asphalt emulsion, which means that after the demulsification of asphalt emulsion there will be more asphalt film that will enfold the cement hydrates and non-hydrated cement grains [62,63]; this will obstruct the cement hydration process and consequently reduce the compressive strength of CA mortar. The modulus of elasticity also decreased with an increase in the A/C due to high asphalt content, and asphalt has a low elastic modulus as compared to hcp; thus, increasing the asphalt content reduces the modulus of elasticity of CA mortar. They also proposed a model which relates the modulus of elasticity and compressive strength of CA mortar to its *V_A_/V_C_* and temperature, as shown in the following equations [60];

$$\sigma =\sigma_{c}\cdot 10^{-b_{\sigma \;˙\;}V_{A}/V_{C}}$$

(3) $$E=\;E_{C}\cdot 10^{-b_{E\;˙\;}V_{A}/V_{C}}$$

where σ and *E* are the compressive strength and elastic modulus of CA mortar, respectively; $\sigma_{c}$ and $E_{C}$ are the compressive strength and elastic modulus of cement mortar, respectively; $b_{\sigma \;}$and $b_{E\;}$ are the positive coefficients of σ and *E* related to the interaction between asphalt and cement [60].

This model was used to predict the mechanical properties of CA mortar with varied *V_A_/V_C_*.

$$A_{\sigma }\;=\;-\;\frac{b_{\sigma ,T1}-b_{\sigma ,T2}}{T_{1}\;-\;T_{2}}\;\cdot \;\frac{V_{A}}{V_{C}}$$

(4) $$A_{E}\;=\;-\;\frac{b_{E,T1}-b_{E,T2}}{T_{1}\;-\;T_{2}}\;\cdot \;\frac{V_{A}}{V_{C}}$$

where $A_{\sigma }$ and $A_{E}$ are the temperature–sensitivity coefficient of the compressive strength and dynamic elastic modulus of CA mortar, respectively; $b_{\sigma ,T1}$ and $b_{\sigma ,T2}$ are the positive coefficients of σ related to the interaction between asphalt and cement at temperatures *T₁* and *T₂*, respectively; $b_{E,T1}$ and $b_{E,T2}$ are the positive coefficients of *E* related to the interaction of asphalt and cement at temperatures *T₁* and *T₂*, respectively [60].

This model was used to predict the mechanical properties of CA mortar at different temperatures.

Equation (4) shows that $A_{\sigma }$ and $A_{E}$ are dependent on *V_A_/V_C_*. When *V_A_/V_C_* increases, the values of $A_{\sigma }$ and $A_{E}$ will also increase, which shows that the properties of CA mortar are more dependent on temperature when the amount of asphalt is high [60]. The calculated data from the two models were consistent with their experimental data; this shows that the proposed models can accurately predict the mechanical properties of CA mortar at varied temperatures and varied *V_A_/V_C_*. These models provided a good and reliable method to predict the mechanical properties of CA mortar. Although the models have some limitations, for instance, when asphalt content in the CA mortar is high, free asphalt may exist within the interlacing structure of the asphalt membrane and cement hydrates; Equation (3) may not be appropriate to relate the mechanical properties of CA mortar with *V_A_/V_C_* in that situation. Additionally, the models did not consider the effect of the loading rate, which is also an important parameter that affects the mechanical properties of CA mortar.

## 4. Use of Additives in CA Mortar Production

Cement is a material with strong adhesive properties used for construction that binds other materials together. Cement binds solid particles such as gravels, aggregate, sand, etc. within a compact structure [64]. Various materials may demonstrate cementitious properties: materials such as natural pozzolans, such as volcanic stuff [65,66], clay [67], and industrial by-products such as slag, fly ash, etc. [68,69,70], can be utilized as a partial substitution for Portland cement in cement-based materials (CBM); these kinds of materials are sometimes referred to as supplementary cementitious materials (SCM). Previous researches have shown that these materials have extensively been used in cement mortars and concrete as partial replacement of Portland cement due to their low cost and accessibility [71], and in the bituminous pavement as mineral filler; the incorporation of these additives in CBM resulted in preserving energy, protecting the environment, and improving the strength and durability of concrete structures in the truculent environment [72,73]. Published research on the influence of volcanic ash in improving performance properties of CBM indicates that it could suitably be used as a partial substitution of cement in CBM [74,75], and when used as filler material in asphalt mixture, it essentially enhances the performance of asphalt pavement and minimizes project cost [76].

Various admixtures in the form of SCMs, industrial by-products, and naturally occurring minerals have been utilized extensively in the production of concrete, cement mortar, and in the bituminous pavement as mineral filler. With regards to CA mortar, there are limited investigations on the utilization of industrial by-products and naturally occurring minerals in the production of CA mortar. However, it was also found in some related works of literature [10,77], that fly ash decreases the viscosity of CA mortar, thereby enhancing its workability. Le et al. [23] reported that substituting cement with any fly ash or ground granulated blast furnace (GGBF) slag improves the overall fresh properties of CA mortar; these materials also prolonged the demulsification process of emulsified asphalt and retarded the hardening process of CA mortar. According to their results, though the mixture with these admixtures suffered slow early strength development as compared to the control mix, a high dosage of fly ash leads to high strength development beyond 28 days. Indeed, favorable particle size distribution, round shape, and appreciative chemical properties of fly ash provided adequate bonding with the asphalt membrane; this helps in providing uniform structure formation in the CA mortar mixture and consequently improves long-term strength development [23]. Their research proves that the utilization of appropriate fly ash in CA mortar improves its fresh and hardened properties, reduces the cost of production, and provides an environmental solution as it utilizes industrial by-products that may rather be used as landfills and eventually cause pollution.

Nanoparticles are among the useful additives with promising influence in improving the performance properties of CBM and bituminous mixtures. Nanoparticles such as nano-SiO₂ and nano-TiO₂ are broadly used to enhance the strength and durability of CBMs [78,79]. Previous researches showed that nanoparticles in CBMs could react with Ca(OH)₂ crystals and generate more C-S-H gel, which occupies the void spaces and enhances the density of the interfacial transition zone [80,81,82]. Therefore, the incorporation of nanoparticles in CA mortar will help in producing more cement hydrates that can absorb the free asphalt in CAB, which will consequently help in reducing the temperature susceptibility of CA mortar. Wu et al. [80] evaluated the flexural strength and compressive strength of CA mortar with and without nano-SiO₂ and nano-TiO₂ at different temperatures, and results of their study indicated that the flexural and compressive strength of CA mortar incorporated with and without nanoparticles decreased when the temperature is increased, but the decreasing rate was found to be reduced in CA mortar incorporated with nanoparticles, especially nano-SiO₂. The existence of free asphalt in CAB is pivotal to the temperature sensitivity of CA mortar; their SEM test results showed that there was less free asphalt in CA mortar with nano-SiO₂, which suggested that the presence of nanoparticles (nano-SiO₂) had a positive effect in reducing the free asphalt, which consequently reduced the temperature sensitivity of CA mortar.

## 5. Conclusions

Cement emulsified asphalt mortar is a major component in the structure of the slab ballastless track. The layer of CA mortar is cast in-between the top track slab and the bottom trackbed of the ballastless slab track structure. It provides support to the track and helps in the load transfer, shock absorption, and vibration dissipation ability of the track system.

The properties of both fresh and hardened CA mortar are highly influenced by the A/C ratio. Asphalt emulsion influences the cement hydration process; likewise, cement hydration affects the breakdown of asphalt emulsion. Therefore, an appropriate A/C ratio is required to achieve the desired strength, damping ability, durability, as well as other mechanical properties of CA mortar. An appropriate A/C is also needed to achieve compatibility among constituent materials of CA mortar.

Air content also has a considerable influence on the properties of CA mortar. Defoamers, mixing speed, and mixing time are used to control the air content of the CA mortar through their influences on the air entrainment and retention.

Further areas of research interests on CA mortar were found to include the incorporation of additives such as SCMs, pozzolans, industrial by-products, and other naturally occurring minerals as partial replacement of cement in CA mortar production; the use of these additives in CA mortar production promotes a greener approach to building construction and sustainable development beside cost-effective benefit and environmental protection. This will ensure broad application of CA mortar as construction material in various aspects of civil engineering.

Additionally, in future research, the influence of the partial substitution of cement with supplementary cementitious materials on improving properties of CA mortar under different environmental and climatic conditions should elaborately be investigated. It is expected that this paper could be useful to those who aim to have a comprehensive understanding of CA mortar composites and performance properties for research purposes.

## Figures and Tables

**Table 1:** Mix proportion for samples preparation [55].

m(E)/m(C) ᵃ (%)	m(E)/m(C) ᵃ (%)	M(W)/m(C) ᵇ
Anionic Emulsifier (JY and ER)	Cationic Emulsifier (JY and PC)	
0	0	0.28
0.6	2	0.28
1.1	4.2	0.28
1.3	4.9	0.28
1.5	5.6	0.28

^a^ Mass ratio of emulsifier to cement, ^b^ mass ratio of water to cement.

## Data Availability

Data sharing not applicable. No new data were created or analyzed in this study. Data sharing not applicable to this article.
